# Thought Leadership on Thrombotic Disorders in South East Asia

**DOI:** 10.7603/s40602-014-0004-2

**Published:** 2014-05-21

**Authors:** Mark Y. Chan

**Affiliations:** National University Heart Centre, Singapore, Singapore

## Abstract

Thrombosis is a leading cause of morbidity and mortality, not only in aging industrialized countries, but also many emerging economies^1^.

Thrombosis is a leading cause of morbidity and mortality, not only in aging industrialized countries, but also many emerging economies^1^. Presently, 110 million patients are at risk of thrombosis worldwide^2^. In Singapore, the incidence of thrombotic diseases parallels that of other developed economies; cardiovascular thrombosis alone caused 27.1% of deaths in 2010, second only to cancer at 29.3%^3^,^4^.

Until recently, research on thrombotic disorders in South East Asia has developed at a far slower pace than the rest of the world, including other parts of Asia.

In this issue of the Journal, we highlight contributions from several South East Asian thought leaders on thrombotic disorders and antithrombotic compounds. At the National University Heart Centre, Singapore, cardiologists have worked hand-in-hand with colleagues from haematology and the basic sciences to develop a thrombosis research program in which we have begun testing variegin, a direct thrombin inhibitor that was discovered in Singapore and is 12 times more potent than bivalirudin^5^. With the How Effective Are Antithrombotic Therapies in Primary Percutaneous Coronary Intervention (HEAT-PPCI) study questioning the true effi cacy of bivalirudin in achieving optimal anticoagulation during percutaneous coronary intervention (PCI)^6^, the search for better parenteral direct thrombin inhibitors has never been timelier. Professor Tan Huay Cheem, director of the National University Heart Centre, Singapore, and his good friend and colleague, Professor Kim Moo-Hyun, addressed the concerns of peri-PCI anticoagulation^7^,^8^ in a debate held last year at the annual National University Heart Centre Atherovenous Thrombosis Symposium. I am also grateful to my friend and colleague, Dr Chee Yen Lin, a consultant haematologist at the National University Cancer Institute, Singapore, who completed her PhD on venous thromboembolism under the mentorship of the venerable Michael Greaves, Aberdeen, UK, immediate past editor of the Journal of Thrombosis and Haemostasis. In this issue of the Journal, Dr Chee has elegantly summarized the role of the novel oral anticoagulants in the management of venous thromboembolism^9^.

Last but most defi nitely not least, Dr Alan Fong and colleagues at the Sarawak General Hospital Heart and Cancer Centre in Kuching, Sarawak, report the results of a single-centre study comparing the performance of novel immunoassay-based pharmacodynamic measures of vitamin K antagonist activity against the international normalized ratio as the gold standard^10^. Dr Fong and colleagues should be congratulated on building impressive infrastructure to conduct dedicated thrombotic research in Kuching, including an early phase clinical trials unit and wet laboratory facilities that include mass spectroscopy-based pharmacokinetic capabilities.

In conclusion, there is now no lack of South East Asian research expertise in thrombotic disorders and antithrombotic compounds. We are optimistic that, with time, thought leaders in our presence will consolidate their efforts to put South East Asia on the world stage of thrombosis research.
